# Heme oxygenase-1 promoter region (GT)n polymorphism associates with increased neuroimmune activation and risk for encephalitis in HIV infection

**DOI:** 10.1186/s12974-018-1102-z

**Published:** 2018-03-06

**Authors:** Alexander J. Gill, Rolando Garza, Surendra S. Ambegaokar, Benjamin B. Gelman, Dennis L. Kolson

**Affiliations:** 1 Department of Neurology, Perelman School of Medicine, University of Pennsylvania, 415 Curie Boulevard, 280C Clinical Research Building, Philadelphia, PA 19104 USA; 2 Department of Botany & Microbiology, Robbins Program in Neuroscience, Ohio Wesleyan University, Delaware, OH 43016 USA; 3 Department of Pathology, University of Texas Medical Branch, Galveston, TX 77555 USA

**Keywords:** Neuroinflammation, Immune activation, Interferon-stimulated genes, Interferon, Heme oxygenase-1, HO-1, HMOX1, HIV-associated neurocognitive disorders, HAND

## Abstract

**Background:**

Heme oxygenase-1 (HO-1) is a critical cytoprotective enzyme that limits oxidative stress, inflammation, and cellular injury within the central nervous system (CNS) and other tissues. We previously demonstrated that HO-1 protein expression is decreased within the brains of HIV+ subjects and that this HO-1 reduction correlates with CNS immune activation and neurocognitive dysfunction. To define a potential CNS protective role for HO-1 against HIV, we analyzed a well-characterized HIV autopsy cohort for two common HO-1 promoter region polymorphisms that are implicated in regulating HO-1 promoter transcriptional activity, a (GT)n dinucleotide repeat polymorphism and a single nucleotide polymorphism (A(-413)T). Shorter HO-1 (GT)n repeats and the ‘A’ SNP allele associate with higher HO-1 promoter activity.

**Methods:**

Brain dorsolateral prefrontal cortex tissue samples from an autopsy cohort of HIV−, HIV+, and HIV encephalitis (HIVE) subjects (*n* = 554) were analyzed as follows: HO-1 (GT)n polymorphism allele lengths were determined by PCR and capillary electrophoresis, A(-413)T SNP alleles were determined by PCR with allele specific probes, and RNA expression of selected neuroimmune markers was analyzed by quantitative PCR.

**Results:**

HIV+ subjects with shorter HO-1 (GT)n alleles had a significantly lower risk of HIVE; however, shorter HO-1 (GT)n alleles did not correlate with CNS or peripheral viral loads. In HIV+ subjects without HIVE, shorter HO-1 (GT)n alleles associated significantly with lower expression of brain type I interferon response markers (*MX1*, *ISG15*, and *IRF1*) and T-lymphocyte activation markers (*CD38* and *GZMB*). No significant correlations were found between the HO-1 (GT)n repeat length and brain expression of macrophage markers (*CD163*, *CD68*), endothelial markers (*PECAM1*, *VWF*), the T-lymphocyte marker *CD8A*, or the B-lymphocyte maker *CD19*. Finally, we found no significant associations between the A(-413)T SNP and HIVE diagnosis, HIV viral loads, or any neuroimmune markers.

**Conclusion:**

Our data suggest that an individual’s HO-1 promoter region (GT)n polymorphism allele repeat length exerts unique modifying risk effects on HIV-induced CNS neuroinflammation and associated neuropathogenesis. Shorter HO-1 (GT)n alleles increase HO-1 promoter activity, which could provide neuroprotection through decreased neuroimmune activation. Therapeutic strategies that induce HO-1 expression could decrease HIV-associated CNS neuroinflammation and decrease the risk for development of HIV neurological disease.

**Electronic supplementary material:**

The online version of this article (10.1186/s12974-018-1102-z) contains supplementary material, which is available to authorized users.

## Background

Heme oxygenase-1 (HO-1) is a sentinel, cytoprotective enzyme that has emerged as a critical effector for limiting oxidative stress, inflammation, and cellular injury within the central nervous system (CNS) and other tissues. The cytoprotective functions of HO-1 have been linked to its degradation of heme and the subsequent generation of carbon monoxide, biliverdin, and bilirubin, which have immunomodulatory and anti-oxidative properties [1]. The expression of HO-1 is rapidly induced during acute cellular injury states in part through transcriptional upregulation by the transcriptional factor Nrf2. Several common genetic variations including (GT)n dinucleotide repeats and single nucleotide polymorphisms (SNPs) within the HO-1 promoter region modulate the level of promoter transcriptional activity, which suggests the likelihood of host variability in executing effective HO-1-driven protective responses to inflammation and cellular injury.

We previously demonstrated that HO-1 protein expression is decreased in the brains of HIV-infected individuals diagnosed with HIV-associated neurocognitive disorders (HAND) and that this reduction of HO-1 is associated with CNS viral load and markers of neuroimmune activation, including type I interferon responses [2]. We also previously identified two potential mechanisms for this loss of brain HO-1 including (i) transcriptional downregulation of HO-1 expression in HIV-infected macrophages, a major CNS HIV reservoir, and (ii) IFNγ-accelerated degradation of HO-1 by the immunoproteasome in astrocytes [3, 4]. Finally, we demonstrated that HIV infection of macrophages not only decreases macrophage HO-1 expression, but that this loss of HO-1 augments neurotoxin production from the infected macrophages [2, 5], thus suggesting that decreased HO-1 expression within the CNS may promote neuronal injury and dysfunction through enhanced neurotoxin production.

Because of the observed relationships between brain HO-1 loss, immune activation, and neurocognitive dysfunction and because of the known associations between HO-1 promoter polymorphisms and increased risk of disease progression in numerous inflammatory diseases, we examined the relationship between HO-1 promoter polymorphisms and CNS disease state in a HIV-infected autopsy cohort. We studied two polymorphisms in the 5′-flanking region of the human HO-1/*HMOX1* gene: a (GT)n dinucleotide repeat that shows length polymorphism, and a single nucleotide polymorphism (SNP), A(-413)T (rs2071746), each of which is known to modulate HO-1 gene transcription under certain conditions. These polymorphisms may also regulate alternative splicing within the 5′ untranslated region of the HO1- gene and contribute to translational regulation [6].

The best characterized of these polymorphisms is the HO-1 promoter region (GT)n dinucleotide repeat polymorphism. The length of this (GT)n repeat typically varies from 12 to 45 repeats with a bimodal or trimodal distribution; the most common alleles have 23 and 30 repeats [7–11]. In studies using luciferase promoter constructs in transfected cell lines, the length of the (GT)n repeat has been demonstrated to modulate HO-1 promoter activity. Specifically, shorter (GT)n repeat lengths produce higher basal promoter activity and higher promoter inducibility in response to various stimuli, including oxidative stress [7, 8]. Additional studies using primary cells and lymphoblastoid cell lines established from subjects with known (GT)n repeat lengths have confirmed these findings [12–14]. Furthermore, other studies have shown that cells with shorter (GT)n repeats are more resistant to oxidative stress-induced apoptosis and have higher oxidative stress-induced HO-1 expression and enzymatic activity [12]. Clinical cohort studies have demonstrated an association between HO-1 promoter region (GT)n repeat length and disease progression in a variety of disease states. For example, shorter (GT)n repeats are associated with better clinical outcomes in patients with emphysema [7], coronary artery disease [15], necrotizing pancreatitis [16], sepsis [17], pneumonia [18], ischemic stroke [19], and rheumatoid arthritis [14], among others. Many of these disease states involve inflammation and oxidative stress, and the association of HO-1 promoter region (GT)n repeat length with disease progression further highlights the potentially significant role for HO-1 in modulating inflammation and oxidative stress in varied pathological conditions, including HIV infection.

To this end, a recent study by Seu et al. demonstrated that shorter HO-1 promoter region (GT)n repeat lengths in HIV-infected African Americans correlated with decreased plasma viral load and soluble CD14, a marker of monocyte/macrophage activation, suggesting a potential link between HO-1 promoter region (GT)n repeat length and HIV disease progression [13]. Moreover, this study demonstrated that fewer (GT)n repeats associated with greater HO-1 protein expression in peripheral blood mononuclear cells and CD14+ monocytes in HIV-infected individuals [13]. The potential role for HO-1 promoter region (GT)n repeat length and neurological disease in HIV infection has not been addressed thus far.

To identify possible associations between the HO-1 promoter region (GT)n repeat length and CNS HIV disease, we quantified the HO-1 promoter region (GT)n repeat lengths in individuals in a large, well-characterized HIV brain autopsy cohort and determined correlations with the pathologic diagnosis of HIV encephalitis (HIVE) and expression of brain tissue-derived markers of immune activation and inflammation. Our data reveal significant associations between the HO-1 promoter region (GT)n repeat length and (i) the pathological diagnosis of HIV encephalitis, (ii) expression levels of type I interferon response genes, and (iii) markers of T-lymphocyte activation in the brains of HIV-infected individuals. These findings suggest that an individual’s HO-1 promoter region (GT)n repeat length serves as genetic determinant of susceptibility to CNS HIV-driven neuropathogenesis associated with neuroinflammation, and they further suggest that HO-1 promoter region (GT)n repeat length genotyping in HIV-infected individuals should be examined as a potential means for identifying an individual’s potential risk for neurocognitive impairment.

## Methods

### Subjects in HIV cohort

A cohort of 442 HIV-positive (HIV+) and 112 HIV-negative (HIV−) subjects was selected from the National NeuroAIDS Tissue Consortium (NNTC) [20] autopsy cohort for genotyping and analysis of RNA expression within dorsolateral prefrontal cortex tissue (DLPFC). This cohort was assembled by the Texas NeuroAIDS Research Center and has been described in a prior report [21, 22]. The 442 HIV+ cases included subjects who had a pathologically confirmed encephalitis (HIVE+; *n* = 92). NNTC site neuropathologists rendered nosological diagnoses of HIVE as guided by the criteria of Budka et al. [23]. See Table 1 for summarized demographic and HIV disease parameter data. Further details on the demographic and clinical data of this cohort were described previously [21, 22].

**Table 1:** Cohort demographics and HIV disease parameters

	Cohort group			
Characteristic	HIV−	HIV+ (no HIVE)	HIV + HIVE	*p* value HIV+ vs. HIVE
Number of subjects	112	350	92	**–**
Age at death, mean ± SD	54.5 ± 16.8	42.8 ± 10.1	41.1 ± 7.7	0.11^a^
Hours postmortem, mean ± SD	15.2 ± 10.9	14.3 ± 13.3	15.5 ± 18.9	0.247^a^
Sex				
Male (%)	67 (60%)	296 (85%)	85 (92%)	0.21^b^
Race				
Caucasian (%)	84 (76%)	209 (60%)	53 (58%)	0.88^b^
African American (%)	24 (22%)	116 (33%)	33 (36%)	
Other/Unknown (%)	3 (2.7%)	25 (7.1%)	6 (6.5%)	
Ethnicity				
Hispanic (%)	22 (20%)	65 (19%)	22 (24%)	0.25^b^
Disease parameters				
Log plasma HIV copies/mL, mean ± SD	–	4.1 ± 1.3	5.1 ± 0.9	< 0.001^a^
Log CSF HIV copies/mL, mean ± SD	–	2.8 ± 1.0	4.3 ± 1.6	< 0.001^a^
Log brain HIV copies/g, mean ± SD	–	3.6 ± 1.2	5.9 ± 1.9	< 0.001^a^
CD4+ lymphocytes/mm^3^, mean ± SD	–	111 ± 168	45 ± 58	0.003^a^

Sample size within disease parameters subgroups are smaller than overall sample size due to the availability of data. *P* value represents comparison between HIV+ (without HIVE) and HIV+ with HIVE subgroups by ^a^Student’s *t* test or ^b^chi-square test. Abbreviations: HIV encephalitis (HIVE), standard deviation (SD)

### DNA isolation and HO-1 promoter genotyping

DNA was isolated from the DLPFC using the DNA Extraction kit (Agilent Technologies, catalog #200600) according to the manufacturer’s instruction, and each sample was standardized to a 20 ng/μl concentration. The sequences of the primers were taken from Seu et al. (2011). The forward primer sequence was labeled at the 5′ end with 6-FAM: 5′-FAM-CCAGCTTTCTGGAACCTTCTG-3′. The reverse primer sequence was unlabeled: 5′-GAAACAAAGTCTGGCCATAGGA-3′. The samples were amplified using the KAPA HiFi PCR Kit (Kapa Biosystems, cat# KK2101) on a thermocycler (Eppendorf Mastercycler RealPlex2) using a touchdown PCR protocol. Samples were initially heated to 95 °C for 10 min, then cycled between 95 °C (20 s) for denaturing, 62 °C (20 s) for annealing, and 72 °C (45 s) for elongating. The touchdown protocol started the annealing temperature at 62 °C and dropped 0.5 °C during each subsequent cycle for the first 15 cycles. The remaining 35 cycles maintained the annealing temperature at 55 °C, followed by a final hold of 72 °C for 10 min. Approximately 1 ng of DNA was used for each sample, and a final primer concentration of 250 nM for both forward and reverse primers was used. PCR products were run on a 2% agarose gel to ensure quality control of amplification of the target sequence. The resulting products were run on the 3130XL Capillary Sequencer (Applied Biosystems) by the Penn Genomics Analysis Core (PGAC) in the Perelman School of Medicine at the University of Pennsylvania (Philadelphia, PA). Sizing was analyzed with GeneMapper (Applied Biosystems) and gated for products 180–300 basepairs in length. All samples were run at least twice from independent PCR reactions to ensure accurate and reproducible sizing and were determined to be accurate within ± 1 GT repeats. A(-413)T SNP (rs2071746) genotyping was performed using TaqMan SNP Genotyping Assay (ThermoFisher, Assay ID: C__15869717_10) per manufacturer’s instructions. Briefly, two SNP target-specific oligonucleotide probes, one for the A-allele and one for the T-allele, with distinct reporter dyes at the 5′ end and a non-fluorescent quencher at the 3′end were used in PCR of genomic DNA with forward and reverse primers covering the region containing the SNP. Exonuclease activity of the DNA polymerase cleaved only probes hybridized to their target sequence, thus releasing the reporter dye and increasing reporter fluorescence. The change in fluorescence of each of the two reporter dyes associated with the two probes determined the presence or absence of the A and/or T SNP allele in the genomic DNA sample.

### Brain dissection and extraction and quantification of brain mRNA

DLPFC was dissected from Brodmann areas 9 or 10. RNA was prepared using the RNeasy Lipid Tissue Mini Kit (Qiagen) as previously described [24, 25]. Briefly, 100 mg of brain tissue was dissected on dry ice and homogenized in a minibead beater. RNA was extracted with chloroform and centrifuged in RNeasy mini spin columns, washed, and eluted. Expression of brain mRNAs was quantified after cDNA synthesis from mRNA samples using Taq-Man Universal PCR Master Mix (Applied Biosystems) by qPCR as previously described [24]. Duplicate qPCR reactions were run and relative mRNA expression was calculated using the ΔΔC_t_ method (compared to GAPDH mRNA expression) using commercially available primer and probe sets from Applied Biosystems: CD19, Hs99999192_m1; CD38, Hs01120071_m1; CD163, Hs01016661_m1; CD68, Hs00154355_m1; CD8A, Hs01555600_m1; GAPDH, Hs99999905_m1; GZMB, Hs01554355_m1; IRF1, Hs00971959_m1; ISG15, Hs00192713_m1: MX1, Hs00182073_m1; PECAM1, Hs00169777_m1; VWF, Hs00169795_m1.

### Quantification of viral loads and CD4 count

In the majority of cases, blood and CSF samples were obtained within 6 months of subject death. Plasma and CSF viral loads were determined with the Amplicor HIV-1 Monitor test v1.1 through v1.5 (*Roche*) and expressed as HIV copies per milliliter. Brain parenchyma HIV RNA was quantified from brain-extracted RNA as previously described and expressed as HIV copies per gram of tissue [24, 25]. Briefly, 1 μg microgram of brain RNA and 1 μM of antisense primer 84R were used in 20 μl reaction (iScript cDNA Synthesis Kit, Bio-Rad Laboratories). Four microliters of cDNA was used for 25 μl quantitative real-time PCR (qPCR) by using JumpStart Taq ReadyMix for Quantitative PCR (Sigma) and SmartCycler (Cepheid). Results were standardized against a known brain secondary standard. CD4+ T-lymphocyte counts were determined by flow cytometry and performed at each NNTC site’s Clinical Laboratory Improvement Amendments (CLIA)–certified, or CLIA equivalent, medical center laboratory.

### Statistics

All group quantifications are expressed as median ± 95% confidence interval. Comparisons of distributions of categorical variables were determined by chi square analysis. Comparisons of continuous variables between groups were performed using the non-parametric Mann-Whitney test or the Kruskal-Wallis test. Analyses of linear trends were performed by Spearman rank correlation. Statistical input support was provided by the Biostatics and Data Management Core, Center for AIDS Research, Perelman School of Medicine, and University of Pennsylvania.

## Results

### HO-1 promoter region (GT)n allele repeat lengths have a distinct modal distribution pattern in African Americans and Caucasians

To determine the potential role for the HO-1 promoter region (GT)n repeat length polymorphism (referred to hereafter as HO-1 (GT)n) in HIV-associated neuropathogenesis, we obtained DNA samples from a well-characterized autopsy cohort from the National NeuroAIDS Tissue Consortium (NNTC) (Table 1) [21, 22]. This cohort included HIV-negative individuals (HIV−, *n* = 112) and HIV-positive individuals with and without the post-mortem diagnosis of HIV encephalitis (HIVE) (HIVE+, *n* = 92; HIV+/HIVE−, *n* = 350). From the DNA obtained, we successfully genotyped the HO-1 (GT) repeat lengths in 549 of 554 (99.1%) of the cohort subjects.

**Fig. 1:**
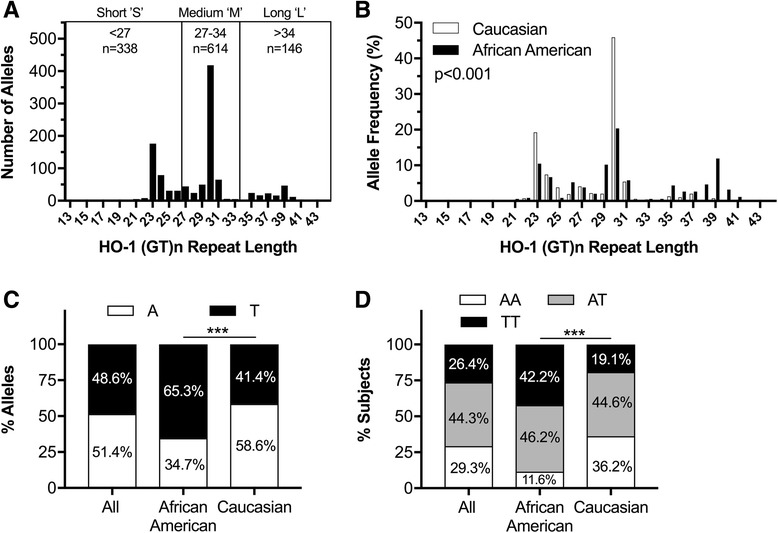
HO-1 (GT)n repeat length and A(-413)T SNP distributions differ between African Americans and Caucasians. HO-1 (GT)n polymorphism allele lengths were determined by PCR and capillary electrophoresis and the HO-1 A(-413)T SNP was determined by TaqMan SNP Genotyping Assay. **a** Histogram of the number of alleles of each HO-1 (GT)n repeat length for the entire cohort (HIV− and HIV+). The trimodal distribution of HO-1 (GTN)n repeat lengths was divided into short ‘S’, medium ‘M’, and long ‘L’ alleles as depicted in the histogram. **b** Histogram of the allelic frequency of each HO-1 (GT)n repeat length in Caucasians and African Americans in the entire cohort. Kolmogorov-Smirnov test revealed a significant (*p* <  0.001) difference in allelic distribution of HO-1 (GT)n repeat lengths between Caucasians and African Americans. **c** Allelic frequencies and **d** genotypic frequencies of the A(-413)T SNP in this cohort, revealing significant allelic and genotypic frequency differences between African Americans and Caucasian subgroups by chi-square (*p* <  0.001). *P* value: ***p <  0.001

In this composite cohort, the HO-1 (GT)n repeat lengths ranged from 13 to 44, with a trimodal distribution of peaks at 23, 30, and 39 repeat lengths (Fig. 1a). This is consistent with previous population cohort studies of HO-1 (GT)n repeat lengths [13]. Based on this trimodal distribution in our cohort, we assigned HO-1 (GT)n repeat length-based allelic genotypes, defined as short ‘S’ (27), medium ‘M’ (27–34), or long ‘L’ (> 34) (GT)n repeat lengths (Fig. 1a). Given our hypothesis that shorter HO-1 (GT)n repeat lengths would be protective against HIV CNS disease, we determined associations with the HO-1 (GT)n polymorphism by two distinct approaches: (i) examining associations with the presence of a short ‘S’ allele, that is having a high HO-1 expressing/inducing promoter, and (ii) examining correlations with the repeat length of an individual’s shortest HO-1 (GT)n allele, that is the length of the repeat in their most expressive/inductive HO-1 promoter. Using these two approaches, we found no significant associations between the HO-1 (GT)n polymorphism and age at death, post-mortem interval, gender, ethnicity, or HIV status (*data not shown*).

However, as previously described [13], we did observe a significant difference in the HO-1 (GT)n allelic distribution between self-identifying Caucasians and African Americans, the two largest racial groups in this cohort (Fig. 1b; *p* < 0.001, Kolmogorov-Smirnov test). The most common HO-1 (GT)n allele length of 30 (‘M’ allele) was observed in both Caucasians and African Americans, with frequencies of 46 and 20%, respectively. In contrast, more African American individuals had long ‘L’ alleles in comparison with Caucasians (37 vs 5%, respectively). These data demonstrate that our autopsy cohort expresses HO-1 (GT)n repeat lengths in a distribution expressed in other clinical population cohorts that have been used to assess effects on clinical- and biomarker-associated outcomes in various disease states.

### The HO-1 promoter region A(-413)T single nucleotide polymorphism has distinct distribution patterns in African Americans and Caucasians

We also genotyped the HO-1 promoter region A(-413)T SNP (rs2071746), which is also reported to affect HO-1 promoter transcriptional activity [11] and affect clinical disease outcomes, albeit to a lesser extent than the HO-1 (GT)n polymorphism [26–28]. We were able to reliably genotype this HO-1 A(-413)T SNP in 553 or 99.8% of the subjects in our cohort and found an overall frequency of the ‘A’ allele of 51.4% and ‘T’ allele frequency of 48.6% (Fig. 1c). In this cohort, the allelic and genotypic frequencies of the A(-413)T SNP were in Hardy-Weinberg equilibrium, although their frequencies differed significantly between Caucasians and African Americans (Fig. 1c, d). African Americans had a higher prevalence of ‘T’ alleles in comparison with Caucasians (65.3 versus 34.7%, respectively). Notably, the A(-413)T SNP and the HO-1 (GT)n repeat length genotypes were not independently expressed in our cohort. Specifically, the ‘A’ SNP allele associated significantly with medium ‘M’ HO-1 (GT)n alleles, whereas the ‘T’ SNP allele associated significantly with the presence of a short ‘S’ or long ‘L’ HO-1 (GT)n allele (*data not shown*).

### HIV-infected individuals with a “short” HO-1 promoter region (GT)n allele have a significantly decreased risk for HIV encephalitis

**Fig. 2:**
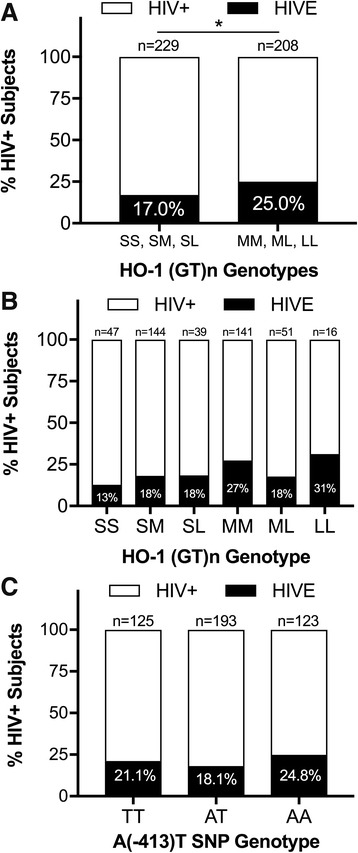
HIV-infected individuals with “short” HO-1 (GT)n repeat lengths have significantly decreased risk for HIV encephalitis. **a** Percentage of HIV+ subjects with HIVE in those with a short ‘S’ HO-1 (GT)n allele (SS, SM, SL) and those without a short ‘S’ allele (MM, ML, LL). HIV+ subjects with a short ‘S’ allele have a significantly reduced risk (odds ratio = 0.62) of having a post-mortem diagnosis of HIVE by chi-square analysis (*p* = 0.04). **b** Percentage of HIV+ subjects with HIVE with each HO-1 (GT)n allele genotype (‘S’ short, ‘M’ medium, ‘L’ long). **c** Percentage of HIV+ subjects with HIVE with each HO-1 A(-413)T SNP genotype groups by chi-sqaure test (*p* = 0.36). *p <  0.05

Given our previous observations demonstrating a significant correlation between reduced HO-1 protein expression within the brain frontal cortex and the diagnosis of HIV encephalitis (HIVE) [2], we first asked whether the HO-1 (GT)n repeat length associated with an altered risk for having pathologically confirmed HIVE. We found that presence of at least one short ‘S’ HO-1 (GT)n allele correlated with a significantly lower risk of HIVE (*p* = 0.04) with an odds ratio of 0.62 (95% CI 0.39–0.98) (Fig. 2a). Notably, the group of individuals with two short alleles, ‘SS’, had the lowest HIVE prevalence (13%) among all HO-1 (GT)n genotypes, while individuals with two long alleles, ‘LL’, had the highest HIVE prevalence (31%; Fig. 2b). There was no correlation between the HO-1 promoter A(-413)T SNP and HIVE prevalence (Fig. 2c). These data support the hypothesis that increased HO-1 promoter inducibility associated with a shorter HO-1 promoter region (GT)n repeat length may be at least partially protective against neuropathogenic processes linked to the development of HIVE.

### Neither the HO-1 (GT)n repeat length nor the A(-413)T SNP correlates with plasma viral load, CNS viral load, or blood CD4 T-cell count in HIV-infected individuals

**Fig. 3:**
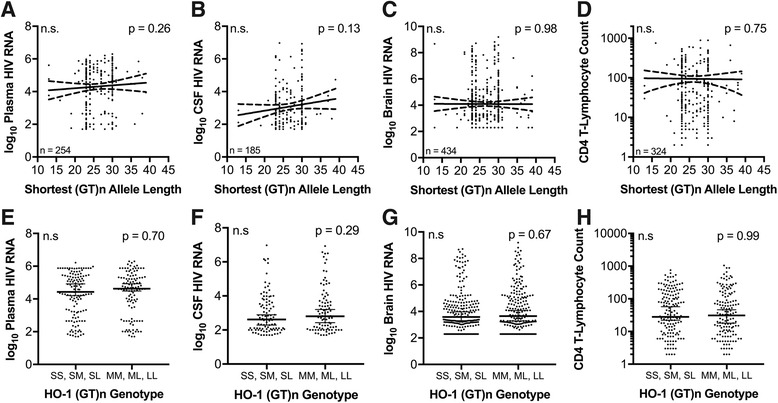
HO-1 (GT)n repeat length does not correlate with plasma or CNS viral load or blood CD4 T-cell count in HIV-infected individuals. Correlations between an HIV+ individual’s shortest (GT)n allele length and **a** plasma viral load (HIV copies/ml), **b** CSF viral load (HIV copies/ml), **c** brain parenchyma viral load (HIV copies/g), and **d** peripheral blood CD4 T-lymphocyte count (cells/mm^3^). Solid lines represent best-fit linear regression models with dashed lines showing 95% confidence bands. Statistical analysis was performed by Spearman rank correlation. **e** Plasma viral load, **f** CSF viral load, **g** brain parenchyma viral load, and **h** peripheral blood CD4 T-lymphocyte count in HIV+ individuals with a short ‘S’ allele (SS, SM, SL) and those without a short ‘S’ allele (MM, ML, LL). Lines and error bars indicate median ± 95% confidence interval. All viral load data is log_10_ transformed. Differences between groups were analyzed by Mann-Whitney test. n.s., not significant

Previous studies suggested that HO-1 may inhibit HIV infection and/or replication [29], providing a potential mechanism by which differential expression of HO-1 could affect HIV pathogenesis, both in the periphery and CNS. In our previous studies, however, we did not observe any effect of induction or suppression of HO-1 expression, or application of HO-1 enzymatic inhibitors, on HIV viral replication in monocyte-derived macrophages [2]. These in vitro findings are consistent with our failure to observe associations between either the HO-1 (GT)n repeat length or the A(-413)T SNP and plasma viral load, cerebrospinal fluid (CSF) viral load, and brain parenchyma viral load, which is presumed to reflect primarily the macrophage/microglial reservoir (Fig. 3 and Figure S1, see Additional file 1). We also failed to observe correlations between either of the HO-1 polymorphisms examined and blood CD4 T-lymphocyte counts (Fig. 3 and Additional file 1: Figure S1). The lack of correlation between either HO-1 polymorphism and viral load in these compartments, or blood CD4 T-lymphocyte count, also were lacking in any subgroup examined: (i) HIV-infected individuals without HIVE, (ii) HIV-infected Caucasians, or (iii) HIV-infected African Americans (*data not shown*). These data suggest that HO-1 does not directly modulate HIV replication in vitro or in vivo.

### Shorter HO-1 promoter region (GT)n alleles correlate with lower brain expression of markers of type I interferon responses and T-lymphocyte activation in HIV-infected individuals

**Fig. 4:**
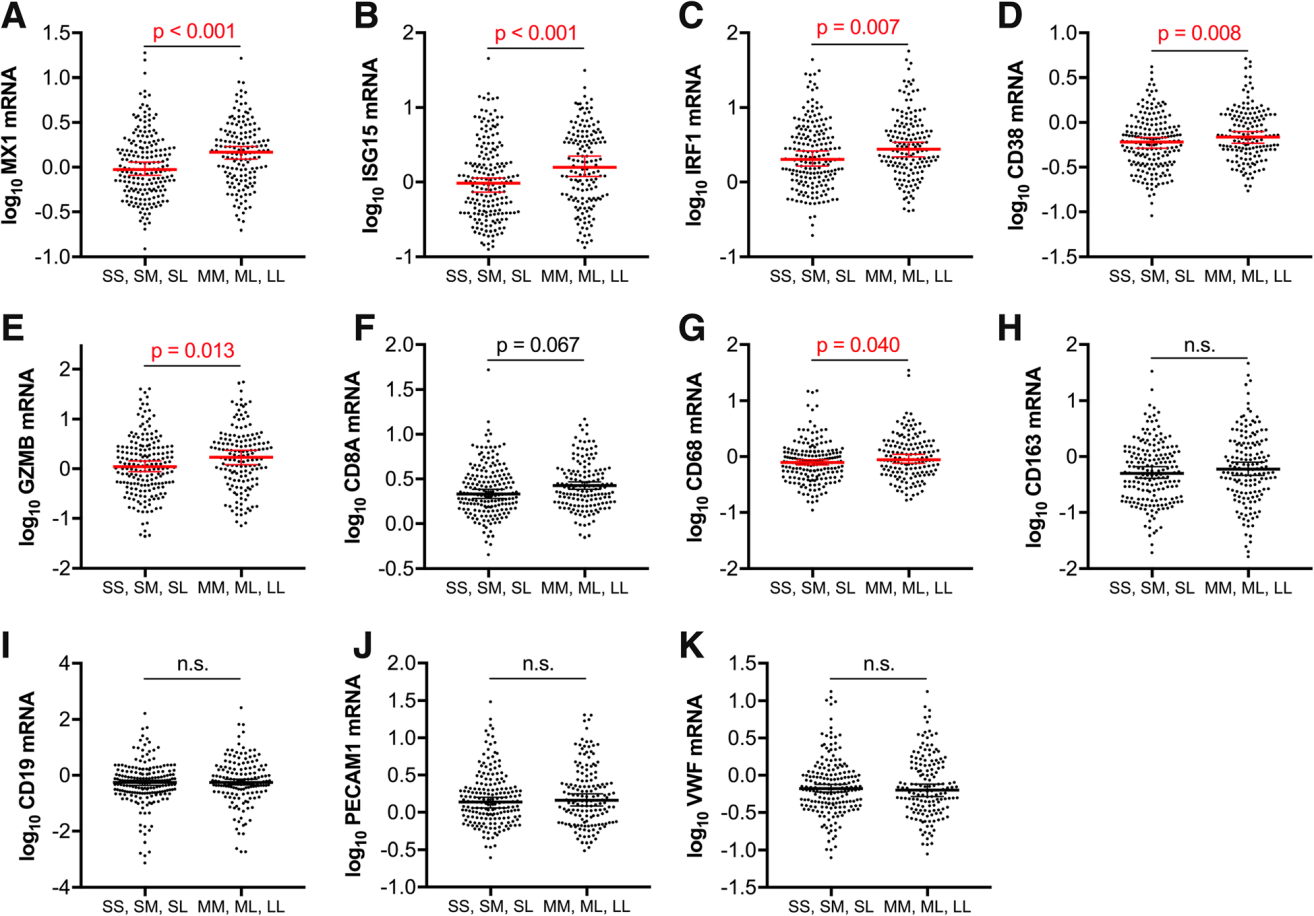
HIV-infected individuals with short ‘S’ HO-1 (GT)n alleles have significantly lower CNS expression of markers of type I interferon responses and T-lymphocyte activation. Prefrotanal cortex RNA expression of neuroimmune markers was compared between HIV-infected subjects (without HIVE) with a short ‘S’ HO-1 (GT)n allele (SS, SM, SL) and those without a short ‘S’ allele (MM, ML, LL). Neuroimmune markers analyzed were **a** MX1, **b** ISG15, **c** IRF1, **d** CD38, **e** GZMB, **f** CD8A, **g** CD68, **h** CD163, **i** CD19, **j** PECAM1, and **k** VWF. Lines and error bars indicate median ± 95% confidence interval of log_10_ transformed RNA expression data. Red median lines and errors bars denote significant differences. Differences between groups were analyzed by Mann-Whitney test. n.s., not significant

**Fig. 5:**
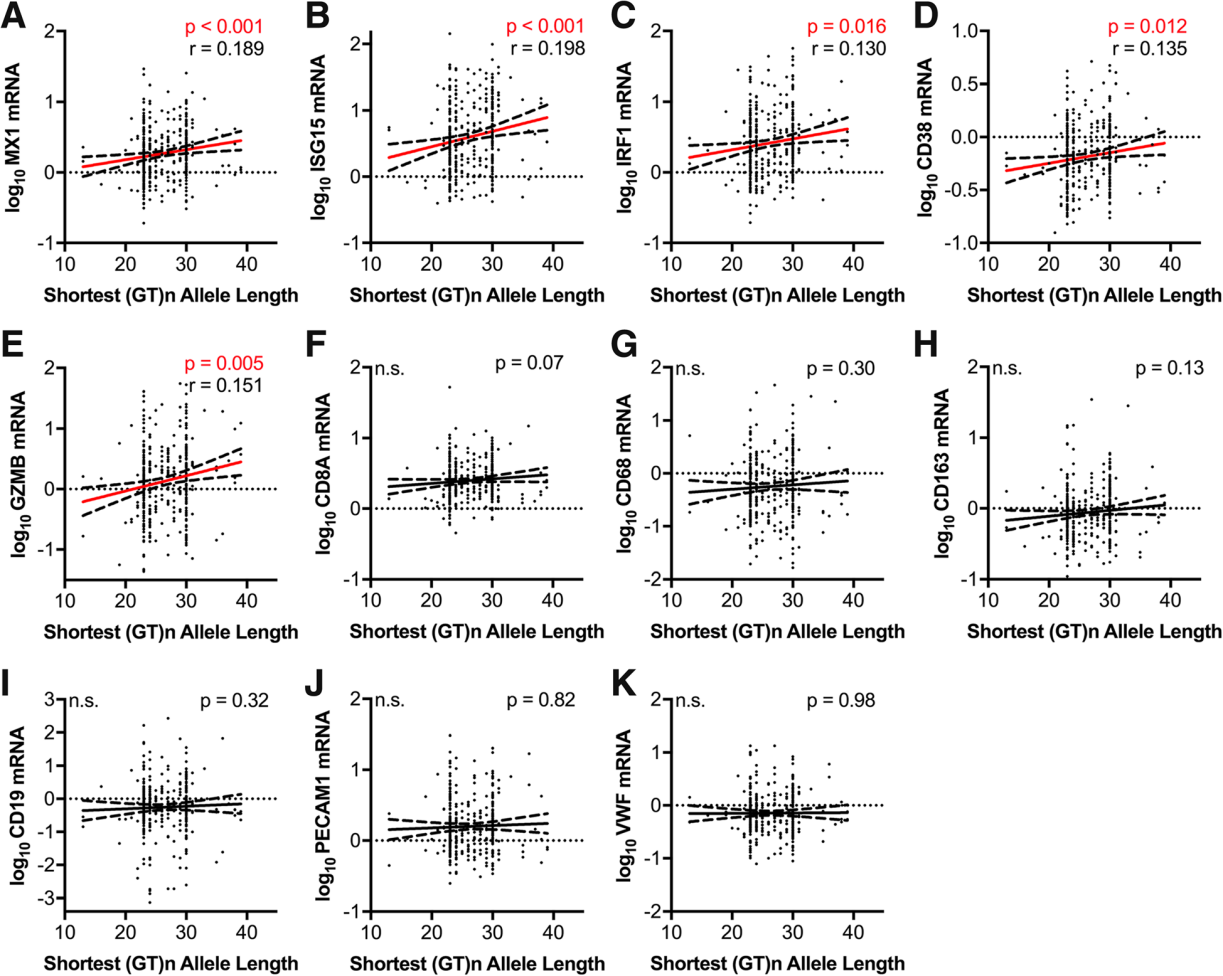
Length of the shortest HO-1 (GT)n allele correlates positively with CNS expression of markers of type I interferon responses and T-lymphocyte activation in HIV-infected individuals. Correlations were determined between the length of an HIV-infected (without HIVE) individuals shortest HO-1 (GT)n allele and the prefrontal cortex expression of the RNA markers **a** MX1, **b** ISG15, **c** IRF1, **d** CD38, **e** GZMB, **f** CD8A, **g** CD68, **h** CD163, **i** CD19, **j** PECAM1, and **k** VWF. Significant associations were determined by Spearman rank correlation. Solid lines represent best-fit linear regression models with dashed lines showing 95% confidence bands. Red regression lines denote significant differences. *r*, Spearman rank coefficient; n.s., not significant

Given the lack of correlation between the HO-1 (GT)n repeat length and markers of systemic HIV disease progression (plasma viral load and CD4 T-lymphocyte count) and the previously identified role for HO-1 in modulating inflammation, we hypothesized that the HO-1 (GT)n repeat length would correlate with CNS markers of immune activation and inflammation. To address this, we quantified RNA expression of selected markers of immune activation and inflammation, including type I interferon (*MX1* and *ISG15*) and macrophage (*CD163*) markers, which we had previously demonstrated to correlate with decreased prefrontal cortex HO-1 protein expression in HIV-infected individuals [2]. In HIV-infected individuals without HIVE, the presence of a short ‘S’ allele (Fig. 4a–f) and the repeat length of the shortest HO-1 promoter region (GT)n allele (Fig. 5a–f) significantly correlated with lower expression of type I interferon-inducible genes (*MX1* and *ISG15*), type I/II interferon-inducible gene *IRF1*, and T-lymphocyte activation genes (*GZMB* and *CD38*, but not *CD8A*). The macrophage/microglial marker *CD68* was significantly lower (*p* = 0.04), in HIV-infected individuals with a short ‘S’ allele (Fig. 4g); however, there was no significant correlation between *CD68* and shortest HO-1 (GT)n repeat length (Fig. 5g). Additionally, presence of a short ‘S’ allele and the shortest HO-1 (GT)n repeat length did not correlate with expression of *CD163*, the B-lymphocyte maker CD19, or with the endothelial markers *PECAM1* and *VWF* (Fig. 4 and Fig. 5h–j). Finally, there were no significant associations between the HO-1 promoter region A(-413)T SNP and expression of any of the neuroimmune markers examined (Figure S2, see Additional file 1).

**Fig. 6:**
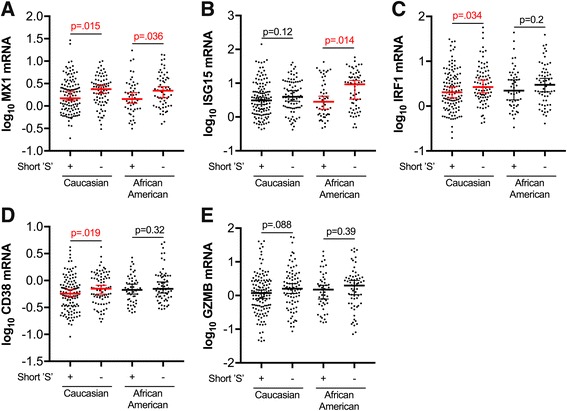
Prefrontal cortex type I interferon responses and T-lymphocyte activation marker expression trends lower in HIV-infected Caucasian and African American individuals with short ‘S’ HO-1 (GT)n alleles. Prefrotanal cortex RNA expression of neuroimmune markers was compared between HIV-infected subjects (without HIVE) with a short ‘S’ HO-1 (GT)n allele (SS, SM, SL) and those without a short ‘S’ allele (MM, ML, LL) in self-identifying Caucasian and African American subgroups. Neuroimmune markers analyzed were **a** MX1, **b** ISG15, **c** IRF1, **d** CD38, and **e** GZMB, those that significantly associated with the HO-1 (GT)n polymorphism in the full HIV+ cohort. Lines and error bars indicate median ± 95% confidence interval of log_10_ transformed RNA expression data. Red median lines and errors bars denote significant differences. Differences in expression between subjects with short ‘S’ alleles and those without ‘S’ alleles in Caucasian and African American subgroups were analyzed by Mann-Whitney test

To confirm that the significant correlations observed between type I interferon and T-lymphocyte neuroimmune markers and the HO-1 (GT)n polymorphism were not being driven by differences in racial subgroup proportions, we compared the expression level of each marker that was significant in the full cohort in Caucasians and African American HIV-infected subjects with and without short ‘S’ alleles (Fig. 6). For *MX1*, the gene most highly significantly different between groups with or without a short ‘S’ allele in the full HIV-infected cohort (Fig. 4a), expression in HIV-infected Caucasian and African American subgroups was significantly lower in individuals with a short ‘S’ allele compared to those without a short ‘S’ allele (Fig. 6a). Moreover, *ISG15* expression was significantly lower in African Americans with a short ‘S’ allele, and *IRF1* and *CD38* were significantly lower in Caucasians with a short ‘S’ allele compared to individuals from the same racial subgroup without a short ‘S’ allele (Fig. 6b–d). For all remaining comparisons in this racial subgroup analysis, expression of each marker was still lower in Caucasians and Africans Americans with short ‘S’ alleles compared to those without short ‘S’ alleles; however, these did not reach statistical significance, potentially as a result of reduced statistical power due to the significantly smaller n in these subgroups (Fig. 6). These subgroup analyses suggest that differences in racial identity (Caucasian or African American) that associate with different HO-1 (GT)n repeat lengths are not driving our observed associations between HO-1 (GT)n repeat length and brain type I interferon and T-lymphocyte neuroimmune marker expression. However, these results do not address or rule out a contributing effect of race on expression levels of these neuroimmune markers within the CNS. Additionally, the significant correlations observed between type I interferon and T-lymphocyte neuroimmune markers and the HO-1 (GT)n polymorphism were highly significant in the full HIV cohort, despite many of these markers positively correlating more significantly and stronger with plasma and CNS viral loads and CD4+ T-lymphocyte count (Table 2). These data suggest that inherent differences in HO-1 promoter activity resulting in higher HO-1 expression may decrease HIV-associated CNS immune activation and neuroinflammation despite significant differences in expression driven by HIV replication and systemic disease progression.

**Table 2:** Neuroimmune markers correlate with CNS and plasma viral load and CD4 T-cell count in HIV infection

RNA	CSF viral load (copies/ml) *n* = 158		Brain viral load (copies/g) *n* = 346		Plasma viral load (copies/ml) *n* = 210		Blood CD4 T-cell count (cells/mm^3^) *n* = 263	
	*r*	*p*	*r*	*p*	*r*	*p*	*r*	*p*
ISG15	0.29	0.0002**	0.21	< 0.0001***	0.46	< 0.0001***	− 0.48	< 0.0001***
MX1	0.29	0.0002**	0.22	< 0.0001***	0.43	< 0.0001***	− 0.42	< 0.0001***
IRF1	0.20	0.0105	0.19	0.0005**	0.31	< 0.0001***	− 0.17	0.0050*
CD38	0.02	0.1243	0.08	0.0317	0.12	0.0155	− 0.09	0.0026*
GZMB	0.21	0.7620	0.17	0.0010**	0.24	0.0155	− 0.20	< 0.0001***
CD8A	0.04	0.6559	0.07	0.1879	− 0.01	0.8860	0.11	0.0823
CD68	0.02	0.8111	0.18	0.1464	0.17	0.0944	− 0.20	0.1353
CD163	0.12	0.0070*	0.12	0.0016*	0.17	0.0005**	− 0.18	< 0.0001***
CD19	− 0.04	0.6468	0.03	0.5175	− 0.13	0.0527	0.26	< 0.0001***
PECAM1	0.06	0.4870	0.03	0.5508	0.07	0.3355	0.02	0.7824
VWF	0.01	0.8518	− 0.01	0.9075	0.08	0.2219	0.01	0.9002

Spearman coefficient (*r*) and *p* value for the correlations between the RNA expression in the frontal cortex of each neuroimmune marker and CSF, frontal cortex (brain), and plasma viral load and blood CD4 T-lymphocyte count. α = 0.01 to correct for multiple comparisons

*p < 0.01; **p < 0.001; ***p < 0.0001

## Discussion

The relatively high prevalence of symptomatic HAND in virally suppressed antiretroviral (ART)-treated HIV-infected individuals underscores the need for adjunctive therapy for improved neuroprotection in HIV treatment strategies [30, 31]. Accumulating evidence suggests that CNS inflammation and oxidative stress can persist within the CNS compartment despite the sustained viral suppression provided by ART [32–34], and this suggests that targeting these processes might offer additional neuroprotection. Our previous studies identified reduced brain HO-1 protein expression in HIV-infected individuals with or without ART treatment, and these studies further showed that this decreased HO-1 expression correlated with increased CNS HIV replication, neuroimmune activation, and cognitive dysfunction (HAND diagnosis) [2]. These results suggest that induction of HO-1 may serve as a protective strategy against neuroinflammation, neuronal injury, and associated neurocognitive impairment in HIV-infected individuals [2, 4, 5].

We now demonstrate a potentially critical link between HO-1 and an individual’s inherent risk for HIV-mediated CNS neuronal injury by identifying the HO-1 promoter region (GT)n repeat length as a risk factor for the development of neuroinflammation and HIV encephalitis (HIVE). We showed that shorter HO-1 promoter region (GT)n repeat alleles significantly correlated with lower brain expression of type I interferon-inducible genes (*ISG15* and *MX1*), type I/II interferon-inducible gene *IRF1*, and T-lymphocyte activation genes (*GZMB* and *CD38*) and a reduced risk of the post-mortem diagnosis of HIVE. Other studies have shown that shorter HO-1 (GT)n repeat lengths are correlated with increased HO-1 gene expression and induction in in vitro model systems and primary cells [7, 8, 12, 13]. While we did observe that African Americans in our cohort have greater HO-1 (GT)n allele length variation that is shifted towards longer repeats compared to Caucasians, as has been previously described [13, 35–37], subgroup analyses demonstrate that these differences in HO-1 (GT)n allele length distributions between these groups are not driving the primary results observed in the full cohort. The reason for increased frequency of longer HO-1 (GT)n alleles in populations of African ancestry is unclear; however, some studies suggest that longer HO-1 (GT)n repeats may be enriched secondary to a protective effect against malaria [35, 36]. In contrast to our observations of significant correlations with shorter HO-1 (GT)n repeat lengths, we did not find any significant correlations between the same markers of HIV-driven neuroinflammation and the A(-413)T SNP in the HO-1 promoter, suggesting this SNP may play a limited or secondary role to that of the HO-1 promoter region (GT)n polymorphism. Importantly, we also did not observe a correlation between the HO-1 (GT)n repeat length and either plasma or CNS viral load or CD4+ T-cell count, suggesting that differential HO-1 transcriptional regulation directly modulates CNS inflammation independent of viral replication and systemic disease progression. Our data thus strongly implicate for the first time a significant CNS effect of HO-1 on HIV-driven neuroinflammation and neurodegeneration, which varies with an individual’s HO-1 promoter genotype, and which itself might be a predictor of risk for development of HAND in HIV infection.

The mechanisms by which HO-1 may modulate HIV neuropathogenesis are not fully apparent, but they likely include effects on cellular oxidation state, glial cell neurotoxin production, and modulation of neuroinflammation. Beyond the direct ability of HO-1 to detoxify free heme, a potent pro-oxidant product of cellular metabolism as well as hemoglobin degradation, HO-1 has been shown to limit cellular and tissue injury in response to non-heme-driven oxidative stress. Adult HO-1-deficient mice express elevated markers of oxidative stress, including oxidized proteins and lipid peroxidation [38, 39] and multiple primary cell lineages (e.g., fibroblasts, vascular smooth muscle, astrocytes) derived from HO-1-deficient mice are highly susceptible to oxidant-induced injury [40–42]. Moreover, in cell culture systems, induction of HO-1 has been shown to protect cells of different lineages (including astrocytes [43, 44] and neurons [45, 46]) from oxidative stress insults [47–50].

We recently demonstrated another mechanism by which HO-1 can specifically modulate neuronal injury, with particular relevance to HIV infection. We showed that loss of HO-1 expression by HIV replication in human macrophages increases their release of glutamate at levels sufficient to induce excitotoxic injury in neurons [2, 5]. Furthermore, this loss of HO-1 protein in HIV-infected macrophages is selective for HO-1 among a number of antioxidant response genes examined [2, 4]. Reduction of HO-1 protein expression in HIV-infected macrophages occurs even with viral strains with limited replication, and ART exposure at CNS-relevant concentrations fails to prevent HO-1 loss and enhanced glutamate release once infection is established [5]. Notably, siRNA knockdown of HO-1 expression or pharmacologic inhibition of residual HO-1 enzymatic activity in HIV-infected macrophages augments glutamate release and associated neurotoxicity, while HO-1 induction decreases glutamate release and associated neurotoxicity [2]. These studies thus suggest that individuals with shorter HO-1 promoter region (GT)n repeat length alleles might be less prone to robust CNS glutamate release and associated neurotoxicity during HIV infection and that CNS induction of HO-1 expression might augment neuroprotective effects of viral suppression by ART.

Additional neuroprotective and cytoprotective effects of HO-1 are likely. In addition to its ability to limit oxidative stress and glutamate production from HIV-infected macrophages, HO-1 can modulate immune activation and inflammation. HO-1 deficient mice exhibit chronic inflammation, as demonstrated by high peripheral white blood cell counts, high CD4+:CD8+ T-lymphocyte ratios, elevated activated CD4+ T-lymphocyte levels, increased monocyte vascular adhesion, increased baseline serum IgM, inflammatory cell tissue infiltrates (particularly in the liver), and elevated splenocyte secretion of pro-inflammatory cytokines in response to endotoxin or anti-CD3/anti-CD28 stimulation [39, 51]. Consistent with an anti-inflammatory role for HO-1, the first identified human patient with HO-1 deficiency demonstrated enhanced systemic inflammation in addition to asplenia, intravascular hemolysis, and systemic vascular endothelial dysfunction, among other symptoms [52, 53]. The anti-inflammatory and immunomodulatory functions of HO-1 have been studied in various immune cells, including those of the myeloid and lymphocyte lineages. In macrophages induction of HO-1 limits expression of pro-inflammatory factors including IL-6, TNFα, inducible nitric oxide synthase (iNOS), and cyclooxygenase-2 (COX-2). In monocytes induction of HO-1 suppresses expression of human leukocyte antigen-DR, CD36, and CD11B [51, 54]. HO-1 also regulates maturation and proper functioning of dendritic cells [55]. In T lymphocytes, HO-1 modulates T lymphocyte-mediated immunity [56], particularly with respect to the suppressive capacity of regulatory T lymphocytes [57]. These data highlight the important roles for HO-1 in modulating immune activation and inflammatory responses and also highlight potential mechanisms by which increased expression of HO-1, through inherent promoter polymorphism differences or exogenous induction, may reduce immune activation and inflammation within the peripheral and CNS compartments.

The decreased neuroimmune activation and neuroinflammation, as indicated by reduced type I interferon responses and T-cell activation markers in the brains of HIV-infected individuals with shorter HO-1 (GT)n alleles, may account for the decreased prevalence of HIVE in these individuals. Subjects with shorter HO-1 (GT)n alleles may have higher basal HO-1 expression and increased induction in response to oxidative stress, inflammation, and other stimuli over the course of their HIV infection, effects that could reduce neuroinflammation, immune activation, and a potential associated risk for developing HIVE. Although HIVE is rarely seen in autopsies from individuals on suppressive ART, the clinical diagnosis of HIV-associated neurocognitive disorders (HAND) remains prevalent in virally suppressed, HIV-infected individuals (~ 15% are functionally impaired) [30, 31]. Moreover, virally suppressed ART-treated individuals express elevated markers of peripheral and CNS inflammation and immune activation, albeit to a lesser extent than untreated HIV individuals [32–34]. Persistent and/or recurrent low-level inflammation and immune activation in ART-treated HIV-infected individuals could contribute to the persistence of HAND [58–61]. A recent retrospective neuropathology study of simian immunodeficiency virus (SIV)-infected pigtailed macaques on ART demonstrated significant prevalence of lymphocyte-dominant inflammation in the CNS [62], suggesting that adaptive immune responses may persist in the CNS in HIV-infected ART-treated individuals and may contribute to neuronal dysfunction and subsequent neurocognitive decline. Moreover, human studies have demonstrated that increased T-lymphocyte activation [63, 64] and type I interferon responses [24, 65, 66] associate with more pronounced neurocognitive dysfunction in HIV-infected individuals.

We hypothesize that shorter HO-1 (GT)n promoter repeat lengths associated with decreased neuroinflammation and neuroimmune activation might decrease the risk of HAND and progression of neurocognitive impairment in HIV-infected individuals on suppressive ART. However, our autopsy study is not ideally suited to assessing the potential contribution of the HO-1 (GT)n promoter repeat length to the development of HAND, given the potential confounding factors of variable ART use and the agonal state of individuals prior to death in autopsy-based cohort studies. However, the data presented in this study build upon our previous data to further support the hypothesis that HO-1 induction would have significant therapeutic benefit in well-suppressed HIV-infected individuals on ART who continue to have elevated markers of immune activation and inflammation and associated disease sequelae. This is additionally built upon previous studies of potentially therapeutic HO-1 inducers and their ability to limit inflammation, oxidative stress, and disease pathology in different disease states [67–69]. To these ends, we are initiating a study to determine whether the presence of short HO-1 (GT)n repeat length alleles associates with HAND, neurocognitive changes over time, and/or with peripheral and CNS biomarkers in a clinically tracked, virally suppressed ART-treated, living HIV patient cohort. Future clinical studies should directly address the potential therapeutic use of HO-1 inducers for neuroprotection against HIV in ART-treated individuals.

## Conclusions

Our data suggest unique modifying risk effects for HIV-induced CNS neuroinflammation and associated neuropathogenesis that are driven by an individual’s HO-1 promoter region (GT)n polymorphism allele repeat length. The presence of shorter HO-1 alleles might provide neuroprotection through decreased neuroimmune activation and neuroinflammation as a result of increased HO-1 promoter activity. Therapeutic strategies that induce HO-1 expression may further decrease HIV-associated CNS neuroinflammation and decrease the risk for development of HIV neurological disease.

## Additional file

Additional file 1: Supplementary Figures. Figures S1 and S2 with corresponding figure legends. (PDF 1377 kb)

## Abbreviations

ART: Antiretroviral therapy
CNS: Central nervous system
CSF: Cerebrospinal fluid
HAND: HIV-associated neurocognitive disorders
HIVE: HIV encephalitis
HO-1: Heme oxygenase-1
NNTC: National NeuroAIDS Tissue Consortium
SEM: Standard error of the mean
SNP: Single nucleotide polymorphism
