# Supplementary material for: Heme oxygenase-1 promoter region (GT)n polymorphism associates with increased neuroimmune activation and risk for encephalitis in HIV infection
Source: J Neuroinflammation. 2018 Mar 6;15:70. doi: 10.1186/s12974-018-1102-z (PMC5838989; doi:10.1186/s12974-018-1102-z)
Supplement: Supplementary file 1 — Supplementary Figures. Figures S1 and S2 with corresponding figure legends. (PDF 1377 kb) [file 12974_2018_1102_MOESM1_ESM.pdf]

Supplementary Figures

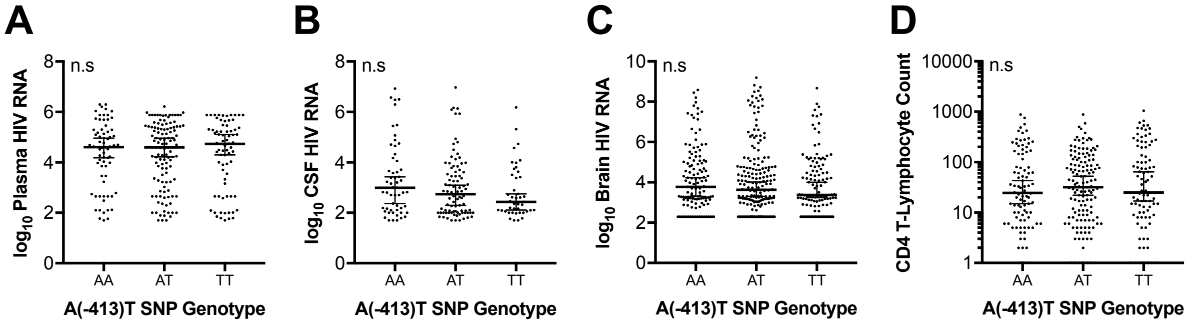

Supplementary Figure 1: **HO-1 A(-413)T SNP does not associate with plasma or CNS viral load or blood CD4 T-cell count in HIV-infected individuals.** (**A**) Plasma viral load (copies/ml), (**B**) CSF viral load (copies/ml), (**C**) brain parenchyma viral load (copies/g), and (**D**) peripheral blood CD4 T-lymphocyte count (cells/mm^3) in HIV+ individuals with AA, AT, and TT A(-413)T SNP genotypes. Lines and error bars indicate median ± 95% confidence interval. Differences between groups were analyzed by Kruskal-Wallis test. n.s. = not significant.

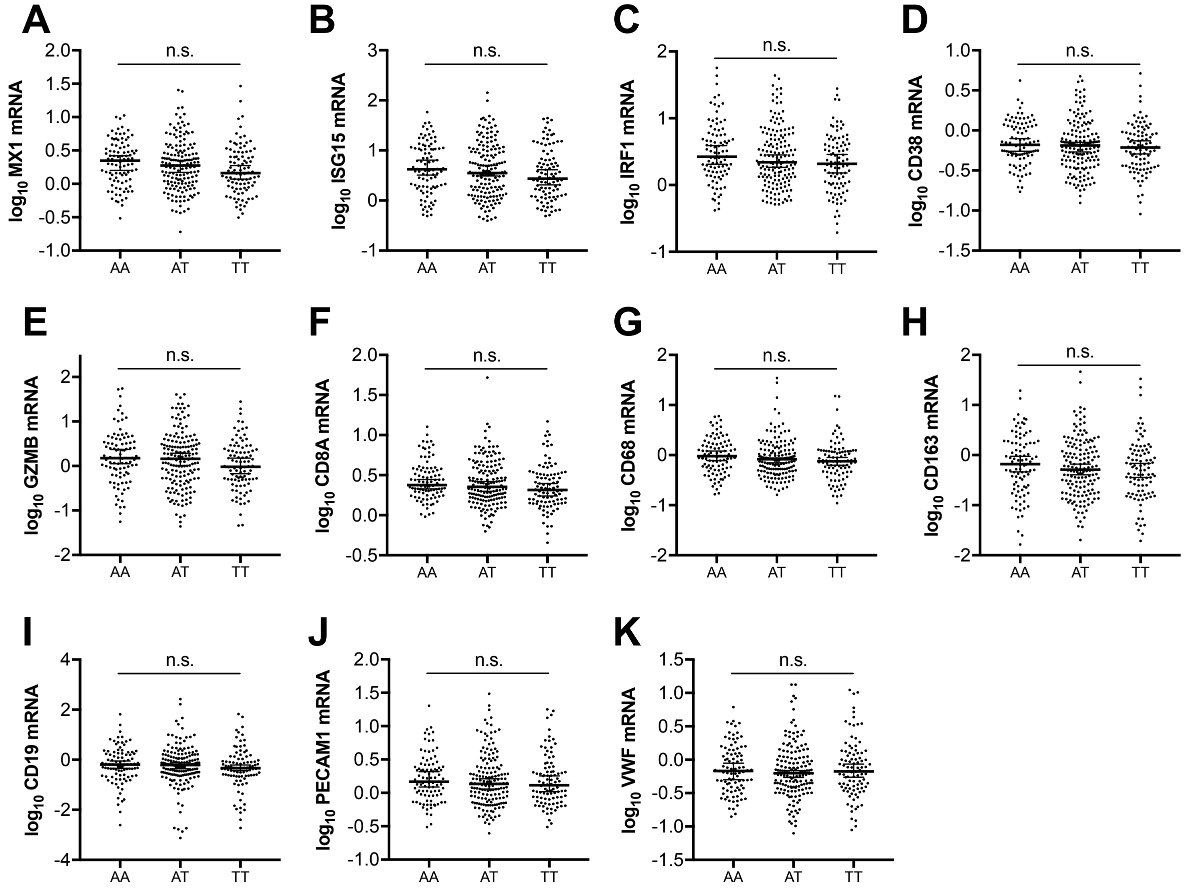

Supplementary Figure 2: **HO-1 A(-413)T SNP does not associate with prefrontal cortex neuroimmune marker expression.** Prefrotanal cortex RNA expression of neuroimmune markers was compared between HIV-infected subjects (without HIVE) with AA, AT, and TT genotypes of the HO-1 promoter region A(-413)T SNP. Neuroimmune markers analyzed were (**A**) MX1, (**B**) ISG15, (**C**) IRF1, (**D**) CD38, (**E**) GZMB, (**F**) CD8A, (**G**) CD68, (**H**) CD163, (**I**) CD19, (**J**) PECAM1, and (**K**) VWF. Lines and error bars indicate median ± 95% confidence interval of $\log_{10}$ transformed RNA expression data. Differences between groups were analyzed by Kruskal-Wallis test.. n.s. = not significant
